# Supplementary material for: Improving antibody language models with native pairing
Source: Patterns (N Y). 2024 Apr 4;5(5):100967. doi: 10.1016/j.patter.2024.100967 (PMC11117052; doi:10.1016/j.patter.2024.100967)
Supplement: Document S1. Figures S1–S3 and Table S1 [file mmc1.pdf]

Patterns, Volume *5*

Supplemental information

Improving antibody language models with native pairing

Sarah M. Burbach and Bryan Briney

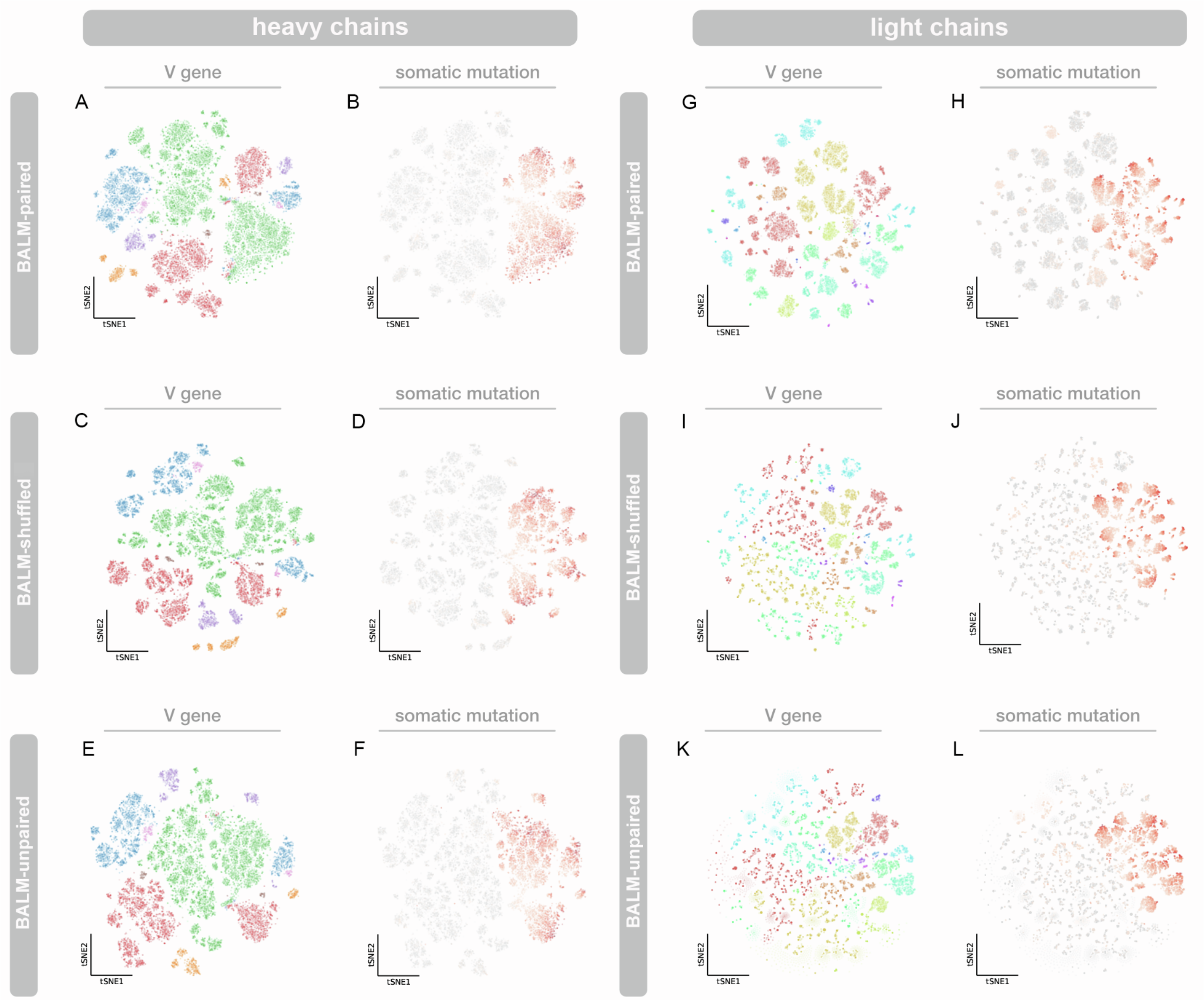

Figure S1. tSNE shows improved clustering of light chains with natively paired sequences. tSNE of final layer embeddings for heavy chains (A-F) and light chains (G-L), colored by V-gene or number of somatic mutations for BALM-paired, BALM-shuffled, and BALM-unpaired. We observe comparable results to the UMAP shown in Figure 2, with v-genes clustering of unmutated light chains being improved by the native pairing in BALM-paired, compared to the more random v-gene clustering observed in BALM-shuffled and BALM-unpaired.

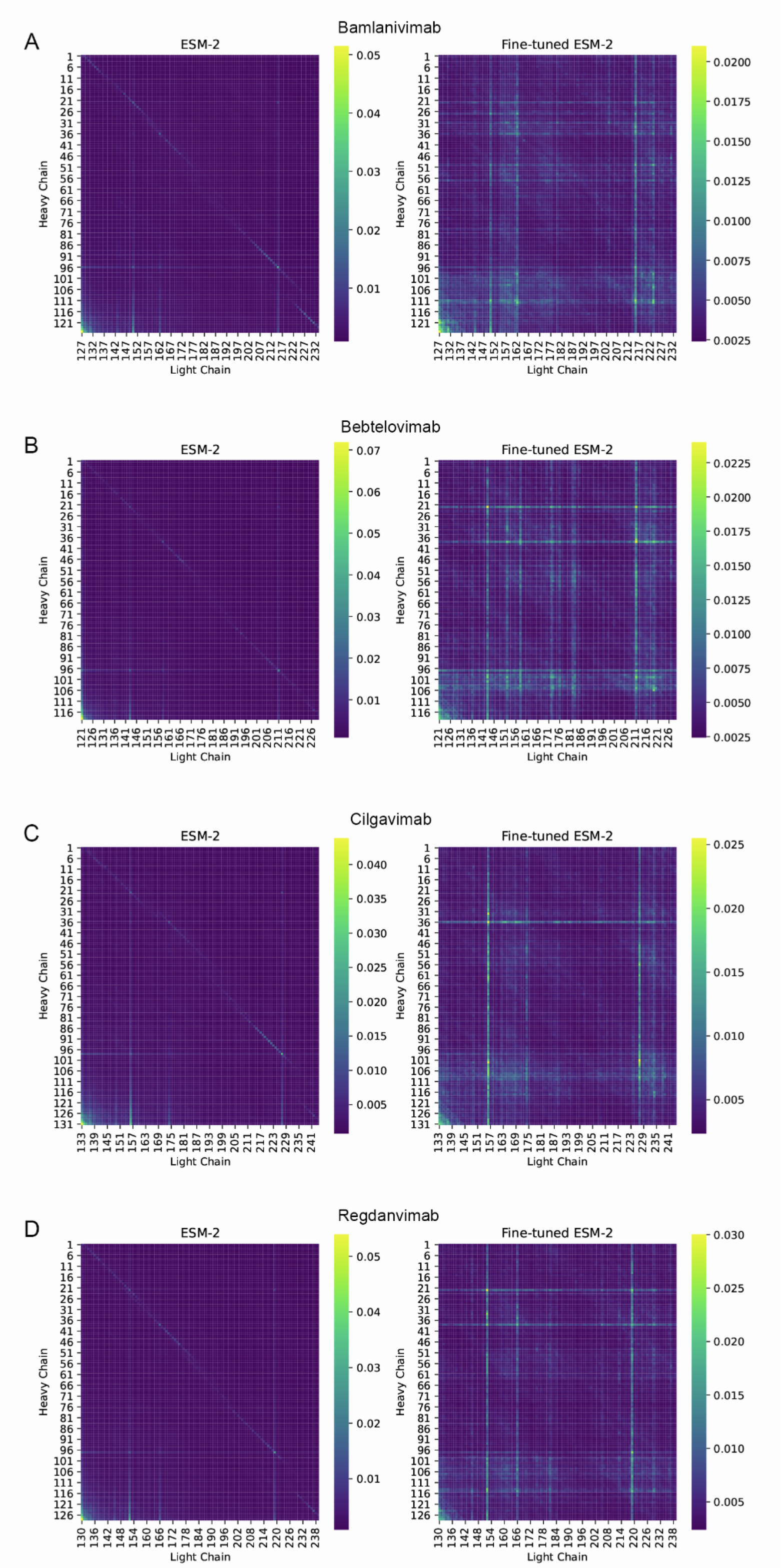

Figure S2. Cross-chain attention for selected therapeutic mAbs. Four therapeutic mAbs against SARS-CoV-2 were processed using base-ESM (right plots) or ft-ESM (left plots): Bamlanivimab (A), Bebtelovimab (B), Cilgavimab (C), and Regdanvimab (D). Cross-chain attention was computed by averaging attention from all heads of each model layer.

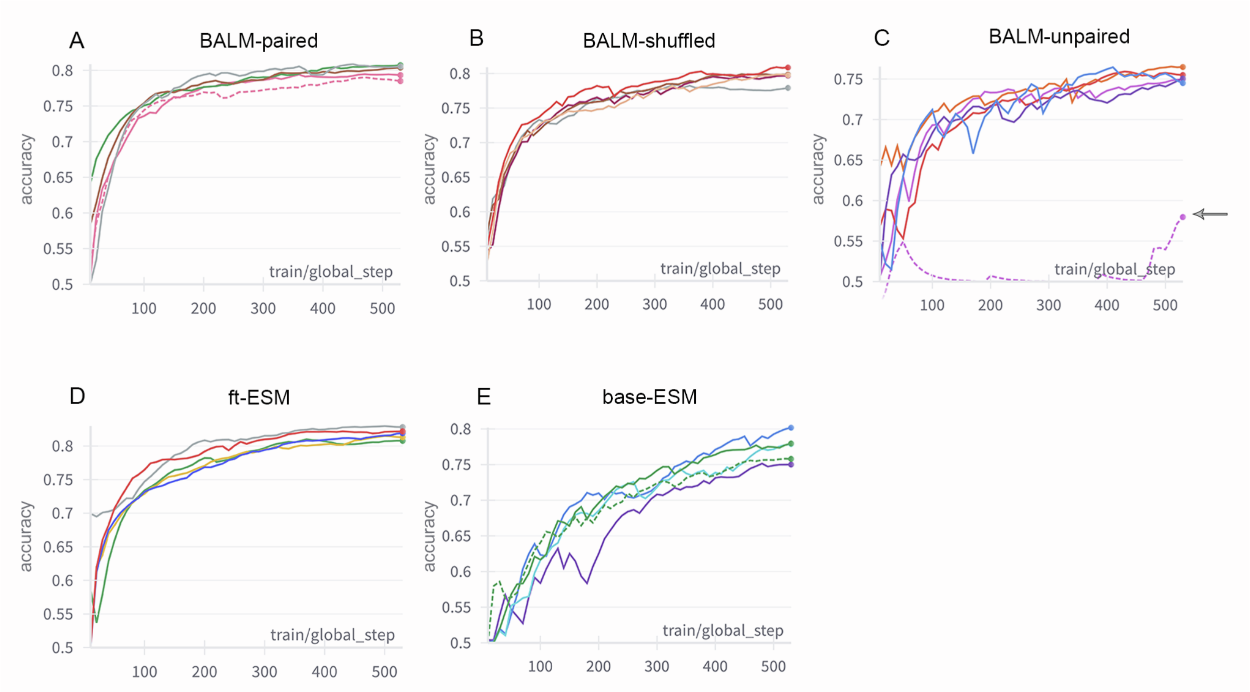

Figure S3. Accuracy training plots for HD vs CoV sequence classification task. Shows the training plots (plotting accuracy vs training steps) for HD vs CoV classification for all five models. We note with an arrow the outlier in BALM-unpaired (C) that was excluded and re-ran with the same dataset, but a different random seed, for Figure 5D.

Table S1. Unpaired classification results for HD vs CoV classification task. Shows results for classification task with the same datasets as the paired HD vs CoV task in Figure 5A, with only heavy chains provided. We observe that model performance of the unpaired / protein models increases and model performance of the paired models decreases compared to the paired classification task. Despite this, ft-ESM remains the highest-performing model, suggesting that mixed-training models are more flexible about the types of data they can accommodate, and therefore are better suited downstream tasks with unpaired datasets than models trained exclusively on paired sequences.

Classification	Model	Accuracy	F1	AUC	AUPR	MCC
CoV vs. Healthy Donor Unpaired Sequences	base-ESM	76.00 (± 0.68)	76.44 (± 0.53)	83.91 (± 0.59)	84.28 (± 0.60)	52.06 (± 1.33)
	BALM-paired	76.66 (± 0.69)	77.12 (± 0.53)	84.84 (± 0.39)	84.90 (± 0.31)	53.45 (± 1.34)
	BALM-unpaired	77.04 (± 0.60)	76.52 (± 0.65)	85.82 (± 0.48)	86.09 (± 0.48)	54.15 (± 1.20)
	BALM-shuffled	77.41 (± 0.90)	78.12 (± 0.79)	85.19 (± 0.89)	85.32 (± 0.86)	55.06 (± 1.77)
	AbLang-H	77.99 (± 0.44)	78.13 (± 0.52)	86.49 (± 0.49)	86.38 (± 0.43)	56.02 (± 0.88)
	AntiBERTy	78.08 (± 0.61)	78.80 (± 0.56)	85.80 (± 0.42)	85.53 (± 0.35)	56.32 (± 1.20)
	ft-ESM	**78.72** (± 0.65)	**78.96** (± 0.45)	**86.72** (± 0.48)	**87.09** (± 0.31)	**57.49** (± 1.26)
