# Supplementary material for: Improving antibody language models with native pairing
Source: Patterns (N Y). 2024 Apr 4;5(5):100967. doi: 10.1016/j.patter.2024.100967 (PMC11117052; doi:10.1016/j.patter.2024.100967)
Supplement: Document S2. Article plus supplemental information [file mmc2.pdf]

Patterns

Article

Improving antibody language models with native pairing

Highlights

- Natively paired models learn immunologically relevant cross-chain features
- Learned cross-chain features result in improved downstream task performance
- Benefits of native pairing cannot be simulated with randomly paired sequences
- Paucity of natively paired sequences can be overcome by training mixed models

Authors

Sarah M. Burbach, Bryan Briney

Correspondence

briney@scripps.edu

In brief

Existing antibody language models are trained using unpaired sequence data, meaning they are unable to learn cross-chain features that are necessary to aid in antibody discovery and development. The authors trained a set of models using natively paired, randomly paired, or unpaired antibody sequence data and show that training with natively paired antibody sequences allows models to learn.

Burbach & Briney, 2024, Patterns *5*, 100967
May 10, 2024 © 2024 The Authors. Published by Elsevier Inc.
https://doi.org/10.1016/j.patter.2024.100967

CellPress

Patterns

CellPress OPEN ACCESS

Article

Improving antibody language models with native pairing

Sarah M. Burbach[1,2,3] and Bryan Briney[1,2,3,4,5,6,*]
[1]Department of Immunology and Microbiology, The Scripps Research Institute, La Jolla, CA 92037, USA
[2]Center for Viral Systems Biology, The Scripps Research Institute, La Jolla, CA 92037, USA
[3]Multi-Omics Vaccine Evaluation Consortium, The Scripps Research Institute, La Jolla, CA 92037, USA
[4]Scripps Consortium for HIV/AIDS Vaccine Development, The Scripps Research Institute, La Jolla, CA 92037, USA
[5]San Diego Center for AIDS Research, The Scripps Research Institute, La Jolla, CA 92037, USA
[6]Lead contact
*Correspondence: briney@scripps.edu
https://doi.org/10.1016/j.patter.2024.100967

THE BIGGER PICTURE Antibodies are used as therapeutics against a variety of human diseases, and their discovery, evaluation, and clinical development would be accelerated by computational models able to infer the characteristics of antibodies directly from their sequence accurately. A human antibody comprises a unique pairing of a heavy chain and a light chain, with both chains contributing to the antigen-binding region of the antibody. Large language models have been used to infer characteristics from an antibody sequence, but these models are usually trained with unpaired sequence data. This means that models cannot learn the cross-chain features necessary to understand structure and function fully. Considering more information during training could further accelerate the clinical development of antibody computational models.

SUMMARY

Existing antibody language models are limited by their use of unpaired antibody sequence data. A recently published dataset of $\sim 1.6 \times 10^6$ natively paired human antibody sequences offers a unique opportunity to evaluate how antibody language models are improved by training with native pairs. We trained three baseline antibody language models (BALM), using natively paired (BALM-paired), randomly-paired (BALM-shuffled), or unpaired (BALM-unpaired) sequences from this dataset. To address the paucity of paired sequences, we additionally fine-tuned ESM (evolutionary scale modeling)-2 with natively paired antibody sequences (ft-ESM). We provide evidence that training with native pairs allows the model to learn immunologically relevant features that span the light and heavy chains, which cannot be simulated by training with random pairs. We additionally show that training with native pairs improves model performance on a variety of metrics, including the ability of the model to classify antibodies by pathogen specificity.

INTRODUCTION

It is estimated that the circulating antibody repertoire is composed of as many as 10^{18} unique antibodies,[1] which surpasses the combined number of unique proteins encoded by all of the genomes of all of the species on Earth by many orders of magnitude.[2] The extraordinary diversity of the human antibody repertoire is produced initially by somatic recombination of germline gene segments.[3] Antibody heavy chains are assembled from variable (V), diversity (D), and joining (J) gene segments. Light chains are assembled similarly, but without D gene segments. This recombination process occurs independently in each B cell, and the resulting antibody is expressed as a dimer of heterodimers, containing two identical heavy chains and two identical light chains. The antigen-binding regions of the antibody, which determine antigen specificity, are each composed of six complementary determining region (CDR) loops: three encoded by the heavy chain and three by the light chain.

Further diversification of antibodies occurs upon exposure to a non-self antigen, when B cells encoding antigen-specific antibodies undergo an iterative affinity maturation process that consists of multiple rounds of clonal expansion, somatic hypermutation (SHM), and antigen-driven selection.[4–6] Through this process, antigenic stimulation of a single naive B cell can produce a clonal lineage of B cells, each expressing an antibody that is related to the parental antibody but which has

This is an open access article under the CC BY license (http://creativecommons.org/licenses/by/4.0/).

accumulated a unique set of somatic mutations. These affinity matured antibodies often contain only a handful of deviations from the original germline recombination, but affinity is typically improved by several orders of magnitude.[7] Following antigen clearance, a subset of B cells encoding affinity matured, antigen-specific antibodies are retained as an immune memory of the encounter,[8,9] which allows rapid response to subsequent exposure and is the primary mechanism of protection for most vaccines. In essence, each person's unique collection of affinity matured antibody genes constitutes a detailed molecular record of all previous pathogen encounters.

The structure and function of a protein is encoded by its amino acid sequence, much as the meaning of a sentence is determined by the order and context of its words. More concisely, sequence determines structure determines function.[10] The conceptual similarity between language and biological sequences inspired the application of language models (LMs) to biological sequence data, to gain a deeper understanding of the language of proteins.[11] LMs trained on general protein sequence data (PLMs), such as HelixFold and ESMFold, have successfully learned information about evolutionary fitness, function, and structure.[12–14] This suggests that the models have learned a deep understanding of the fundamental properties of amino acids and the importance of the order and context in which they occur. Applying PLMs to antibody sequences yielded some success, but PLMs generally exhibited only a cursory understanding of antibodies that did not extend beyond "obvious" features such as germline gene use.[15,16]

Antibody-specific LMs (AbLMs), which use essentially unmodified LM or PLM model architectures but are trained using antibody sequence data, have learned features such as SHM[16–18] and are substantially better than PLMs at antibody sequence infilling.[16] These results indicate that AbLMs possess a more sophisticated understanding of features that differentiate antibodies from the general protein space and provide a strong argument for training specialized AbLMs instead of repurposing pretrained PLMs. However, AbLMs still have much room for improvement.

ESMFold and HelixFold demonstrate that existing model architectures can support powerful biological LMs. Thus, the primary factors impeding AbLM development are instead related to the lack of suitable training data at a sufficient scale. First, existing transformer-based AbLMs were trained using unpaired antibody sequences. This was by necessity rather than design; the far lower cost of generating unpaired sequences means there are orders of magnitude more unpaired than natively paired antibody sequences available.[19,20] Nevertheless, AbLMs trained using only unpaired data cannot learn cross-chain features that encode important information about antibody structure and function. Second, publicly available antibody datasets are skewed toward a relatively small number of disease states, including autoimmunity, cancer, and infectious diseases such as HIV, influenza, and severe acute respiratory syndrome coronavirus 2 (SARS-CoV-2). This produces AbLMs with a parochial view of the antibody repertoire rather than a complete understanding of antibody diversity.

A recently published dataset of ~1.6 million natively paired human antibody sequences[21,22] provides an opportunity to assess the value of training an AbLM with natively paired data. This unique dataset from Jaffe et al., which is the largest publicly available collection of natively paired human antibody sequences, was compiled using circulating B cells from healthy adult donors to produce a minimally skewed representation of the baseline human antibody repertoire. The Jaffe dataset is much smaller than the unpaired datasets used to train existing AbLMs, however, and it is unlikely that the training advantages of native pairing are sufficient to overcome this massive difference in scale. Thus, the primary goal of this work is to determine whether and to what extent an AbLM can be improved by training with natively paired antibody sequence data rather than unpaired sequence data.

To accomplish this, we trained three baseline antibody language model (BALM) variants using identical training datasets except for their inclusion of natively paired sequences (BALM-paired), inclusion of randomly paired sequences (BALM-shuffled), or exclusion of pairing information (BALM-unpaired). BALM-paired performs substantially better than BALM-shuffled and BALM-unpaired across a variety of metrics, with notable improvements in the information content of light-chain embeddings. We further demonstrate that the improved performance of BALM-paired is linked to its ability to learn features that span the heavy and light chains of natively paired antibodies. We additionally fine-tuned an ESM (evolutionary scale modeling)-2 model (ft-ESM) with the same natively paired sequences, to demonstrate a potential middle-ground approach for training a highly performant model despite the limited availability of natively paired data. Finally, we show that these paired models exhibit improved performance over unpaired models on three antibody specificity classification tasks.

RESULTS

Training a BALM

BALM-paired, BALM-shuffled, and BALM-unpaired use a slightly modified RoBERTa-large architecture. An encoder-only architecture was chosen to enable the production of informative sequence embeddings that can be used for downstream tasks, such as specificity classification, and to align with existing protein and antibody LMs. At the time of model training, the notable outlier that was trained as an encoder-decoder was ProtT5. However, ProtBERT performed nearly as well as ProtT5-XL on several downstream tasks despite the fact that the ProtT5-XL model was nearly 10 times larger (3 billion vs. 420 million parameters) and was trained using UniRef50 (ProtBERT was trained using the noisier and more redundant UniRef100). Therefore, an encoder-only architecture was also chosen to enable more efficient training.

Models were trained with a masked language model (MLM) objective on the same Jaffe dataset of 1,335,854 antibody sequence pairs.[22] BALM-paired was trained on the original natively paired sequences, and BALM-shuffled was trained on paired sequences for which the pairing of heavy and light chains was randomized. BALM-unpaired was trained on the light and heavy chains separately, with only one chain per input. To equalize training between the models, BALM-unpaired was trained using a batch size of 512, which is twice that of BALM-paired and BALM-shuffled, at 256.

CellPress
OPEN ACCESS

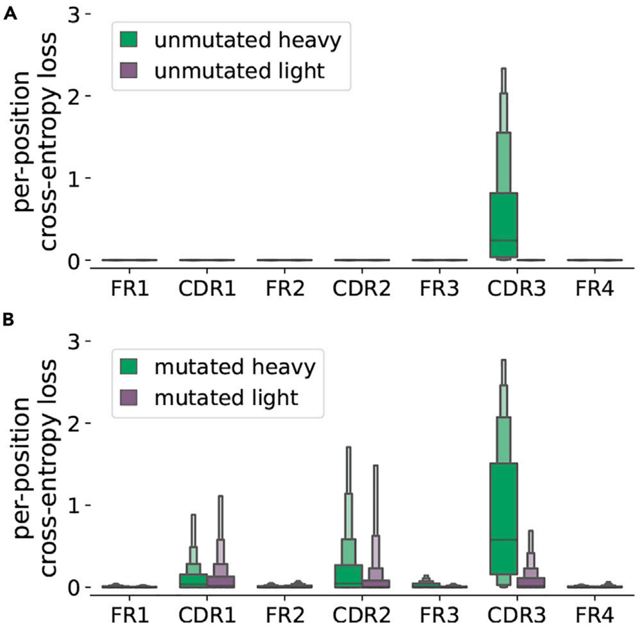

Figure 1. Per-position CEL of BALM-paired

(A and B)The per-position CEL was calculated by iteratively masking each position and predicting the masked residue with BALM-paired using either unmutated (A) or mutated (B) test sequences. For each sequence, the median CEL was computed for each FR or CDR, and the distribution of median values is shown using a letter value plot.[24]

BALM rapidly learns germline antibody features

The combinatorial diversity of antibody recombinants (i.e., the diversity provided by the selection of individual V, D, and J genes for recombination) is relatively small compared to the diversity contributed by nontemplated addition and deletion at recombination junctions.[23] Thus, it is expected that AbLMs will learn germline-encoded features more readily than the more complex patterns inherent in nontemplated regions. To assess this on BALM-paired, we separately analyzed the per-position cross-entropy loss (CEL) of mutated or unmutated sequences (Figure 1). By grouping sequence positions into their corresponding framework region (FR) or CDR, we observed much weaker model performance in the untemplated CDR3s of unmutated sequences. In addition, we observed moderately lower model performance in all of the regions of mutated antibody sequences, which contain nontemplated somatic mutations distributed throughout the sequence. Antibody mutations are clustered in CDRs, and BALM-paired performs substantially less well in the CDRs of mutated sequences compared to the relatively less mutated FRs.

Native pairing preferentially improves light-chain embeddings

Previously reported AbLMs AntiBERTa[18] and AbLang[16] have shown that clustering the output embeddings of these models can group antibody sequences according to V gene use and SHM. Despite both of these AbLMs being trained on datasets that include light chains, only heavy-chain embeddings were analyzed. Using a test dataset of 20,000 natively paired antibody sequences, we analyzed the output embeddings of BALM-paired, BALM-shuffled, and BALM-unpaired. As described previously,[16,18] embeddings from the final transformer layer were averaged along the input length dimension and a uniform manifold approximation and projection (UMAP) representation was computed.[25,26] Because BALM-paired and BALM-shuffled output embeddings include both heavy and light chains, we extracted a subset of the embeddings that contains only the positions corresponding to a single chain (either heavy or light) before averaging over the length dimension. This allows us to directly compare the embeddings produced by the paired models and BALM-unpaired.

Heavy-chain embeddings from BALM-paired, BALM-shuffled, and BALM-unpaired clustered similarly, grouping sequences primarily by mutation and secondarily by V gene (Figures 2A–2F). This mirrors results seen with AntiBERTa[18] but differs slightly from AbLang,[16] for which output embeddings cluster primarily by V gene and secondarily by mutation. However, clustered light-chain embeddings of the models were quite different (Figures 2G–2L). Although BALM-unpaired embeddings of mutated light-chain sequences form reasonably well-defined V gene clusters, unmutated light-chain embeddings were essentially randomly dispersed (Figures 2K and 2L). The same pattern was observed with BALM-shuffled, with unmutated light-chain embeddings appearing to be essentially randomly dispersed (Figures 2I and 2J). In contrast, the clustered light-chain embeddings produced by BALM-paired were similar to heavy chains, segregating sequences primarily by mutation and secondarily by V gene (Figures 2G and 2H). Similar clustering patterns were observed with t-distributed stochastic neighbor embedding (t-SNE) representations (see Figure S1), which verifies that these patterns are not an artifact of the dimensionality reduction method. Given that this improvement in light-chain clustering is only present for BALM-paired and not BALM-shuffled, this suggests that BALM-paired is learning cross-chain features present only in natively paired sequences and that these features preferentially improve light-chain embeddings.

Paired model improvements are driven by learning cross-chain features

We next sought to more deeply investigate the ability of BALM-paired to learn features that span both antibody chains. From our test dataset, we selected all of the sequence pairs containing at least 3 mutations in each of the heavy and light chains. All of the mutated heavy chain positions were masked, and BALM-paired was asked to predict the masked residues when the heavy chain was paired with (1) the natively paired light chain, or (2) a germline-reverted version of the light chain in which all of the mutated light-chain residues were reverted to germline (Figure 3A). For comparison, BALM-unpaired was also asked to predict the same masked residues given only the unpaired heavy-chain sequence. Because only mutated positions were masked, predictions of the germline-encoded residue were always incorrect. For BALM-paired we noted a large reduction in CEL when the masked heavy chain was paired with the native (mutated) light chain (Figure 3B), indicating that native pairing improved model performance through cross-chain learning. BALM-paired considered the correct (mutated) residue ~4-fold more likely when the masked heavy chain was paired with the native light chain (6.7 vs. 1.7), and also considered incorrect but

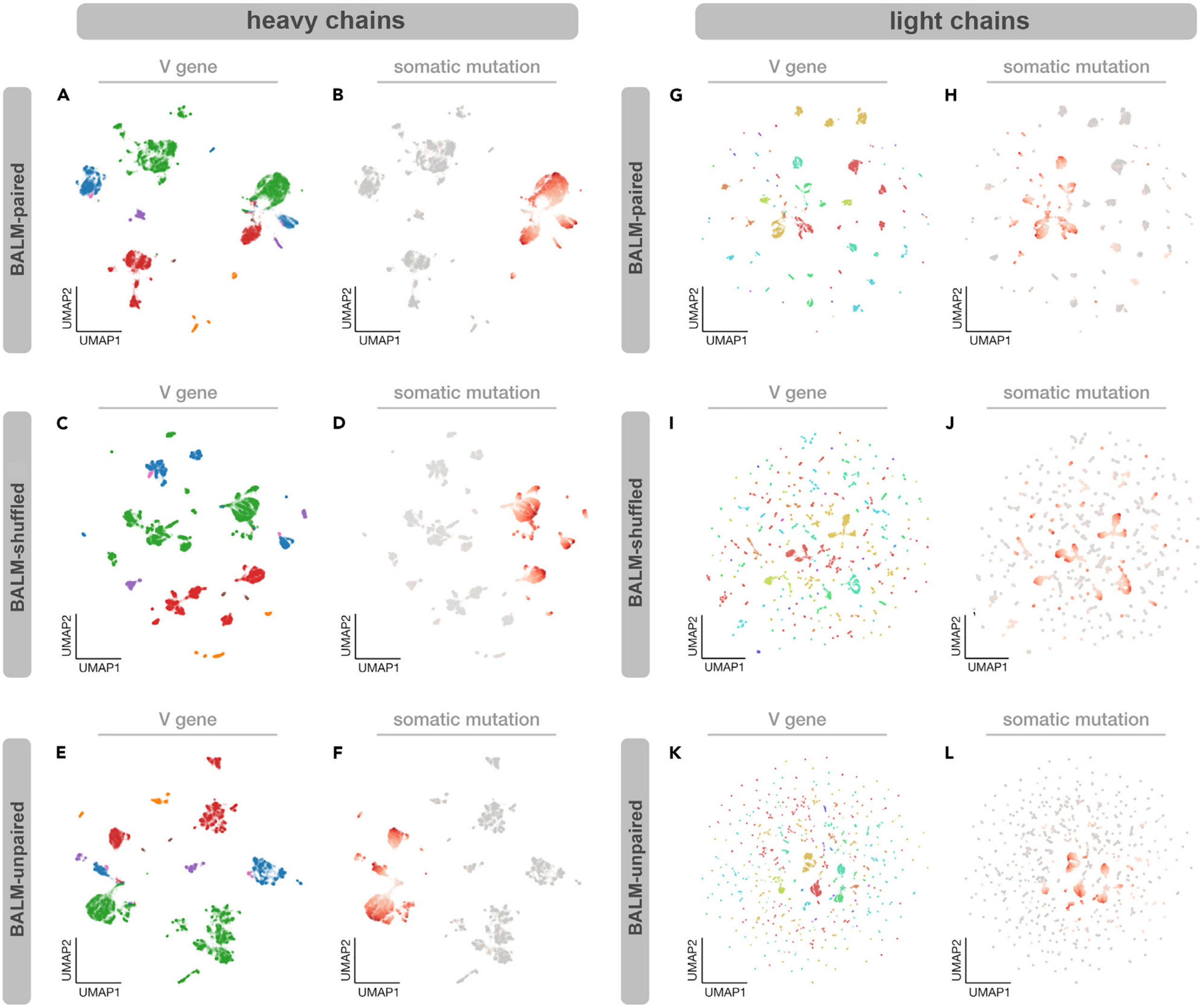

Figure 2. Training with natively paired sequence data improves light chain embeddings

UMAP of final layer embeddings for heavy chains (A–F) and light chains (G–L), colored by V gene or number of somatic mutations, for BALM-paired (top row), BALM-shuffled (center row), and BALM-unpaired (bottom row).

nongermline residues about twice as likely when the masked heavy chain was paired with the native (mutated) light chain (16.7 vs. 8.9), indicating that the model is learning patterns of somatic mutation rather than memorizing specific mutations (Figure 3C). Results from the reciprocal experiment, in which light-chain mutations were masked and paired with native or germline-reverted heavy chains (Figures 3D and 3E), were even more striking: native pairing increased the likelihood of the correct (mutated) residue by >6-fold (7.3 vs. 1.1) and the likelihood of any non-germline residue by nearly 5-fold (11.9 vs. 3.1). To verify that native pairing, and not simply the presence of any paired chain during training, was responsible for the better performance of BALM-paired, we performed the same experiment using BALM-shuffled. The performance of BALM-shuffled was indistinguishable from BALM-unpaired when mutation-masked sequences were paired with either the mutated or germline-reverted partner chain, demonstrating that the features learned by BALM-paired are indeed specific to natively paired antibody sequences.

ft-ESM2 with natively paired sequences

Upon observing the training benefit of natively paired sequences, but recognizing the limited availability and extremely high cost of generating natively paired antibody datasets, we were motivated to evaluate whether a general protein LM could learn similar cross-chain features by fine-tuning with natively paired antibody sequences. This could decrease the amount of natively paired sequences required to construct a competitive model by transferring general protein knowledge and requiring the fine-tuned model to learn only those features that are unique to antibodies. We fine-tuned the pretrained 650-million parameter ESM-2 model[14] (ft-ESM) with an MLM objective on the same dataset of 1,335,854 natively paired antibody sequences used to train BALM-paired.[22]

CellPress
OPEN ACCESS

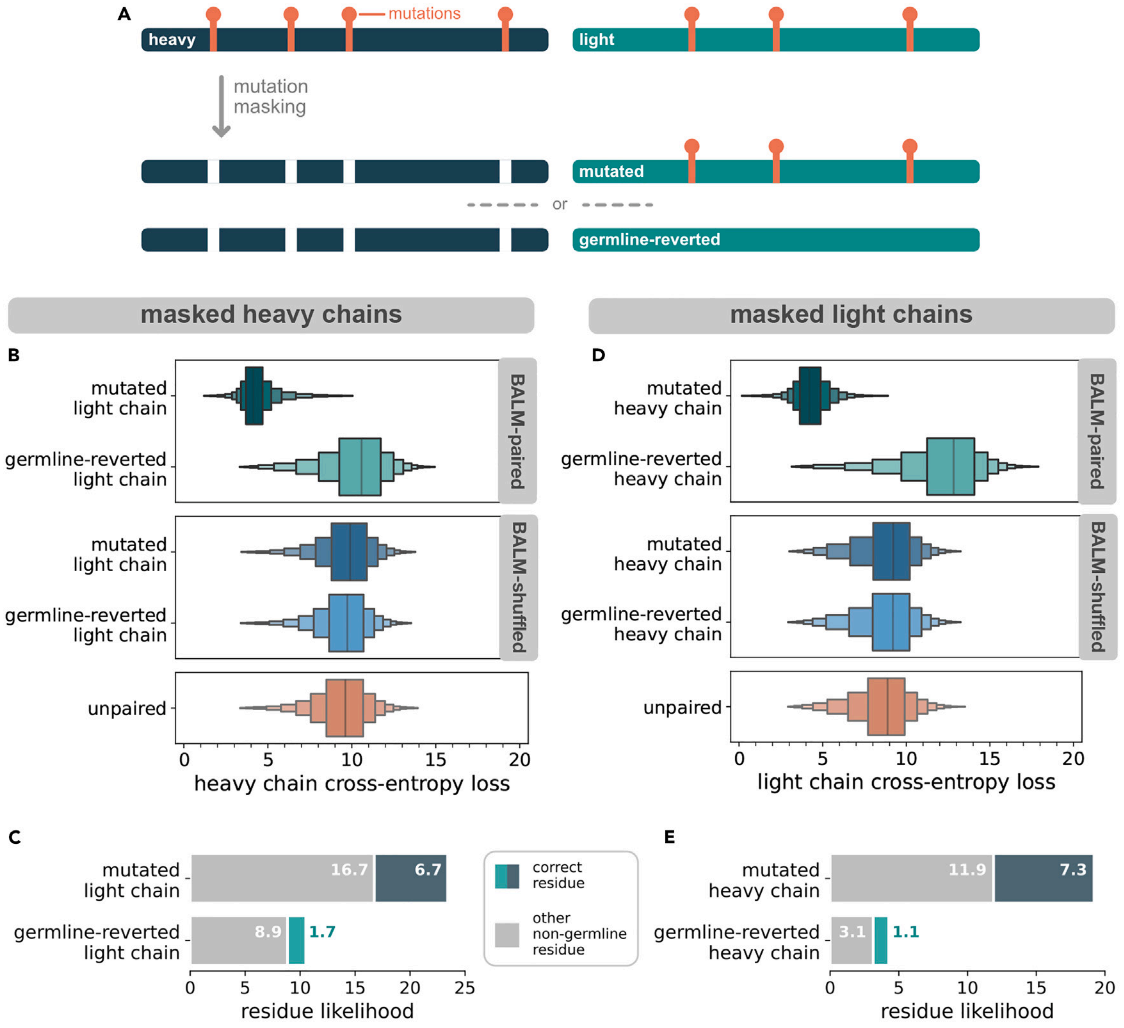

Figure 3. CEL of masked mutations in light and heavy chains

(A) Schematic of the mutation masking process, in which the mutated positions in a single chain are masked and the masked chain is paired with either the native (mutated) partner chain or a germline-reverted variant of the partner chain.

(B) CEL of masked mutations in heavy chains, when paired with mutated light chain and germline reverted light chain for BALM-paired and BALM-shuffled, and alone for BALM-unpaired.

(C) Likelihood (model output probabilities, multiplied by 100) assigned by BALM-paired to the correct masked heavy-chain residue (blue: mutated light chain, green: germline-reverted light chain) or to any other nongermline residue (gray). Likelihood values are the average of all of the masked positions across all test sequences.

(D) CEL of masked mutations in light chains when paired with mutated heavy chain and germline reverted heavy chain for BALM-paired and alone for BALM-unpaired.

(E) Likelihood assigned by BALM-paired to the correct masked light-chain residue (blue: mutated heavy chain, green: germline-reverted heavy chain) or to any other nongermline residue (gray). Likelihood values are the average of all of the masked positions across all of the test sequences.

Learned cross-chain features are immunologically relevant

To assess the cross-chain features being learned by our natively paired models, 1,000 sequences were randomly selected from the test dataset and attention values for all of the attention heads in the final layer of each model were extracted. The attention values were filtered to produce a cross-chain attention matrix containing only position pairs that span both antibody chains. This matrix was averaged by sequence position and each position was categorized by antibody region (CDR or FR) to calculate the percentage of cross-chain attention directed toward CDRs compared to FRs.

We first sought to determine which regions of the antibody sequences were the focus of model attention. For both models trained on natively paired antibodies (ft-ESM and BALM-paired), we observed heightened cross-chain attention in CDRs, with ft-ESM paying 2.05 times more attention to CDRs than FRs and BALM-paired devoting 1.44 times more attention (Figure 4A). BALM-unpaired and base-ESM devote approximately equal cross-chain attention to CDRs and FRs. Notably, the cross-chain attention patterns of BALM-shuffled match the unpaired models, demonstrating once again that native pairing, rather than simply the presence of any random paired chain

A

Model	CDR Percentage	CDR Attention (%)	Ratio
BALM-unpaired	22.43 (± 1.45)	21.68 (± 2.17)	0.97
base-ESM		22.53 (± 1.18)	1.00
BALM-shuffled		23.51 (± 2.15)	1.05
BALM-paired		32.30 (± 4.43)	1.44
ft-ESM		46.08 (± 4.65)	2.05

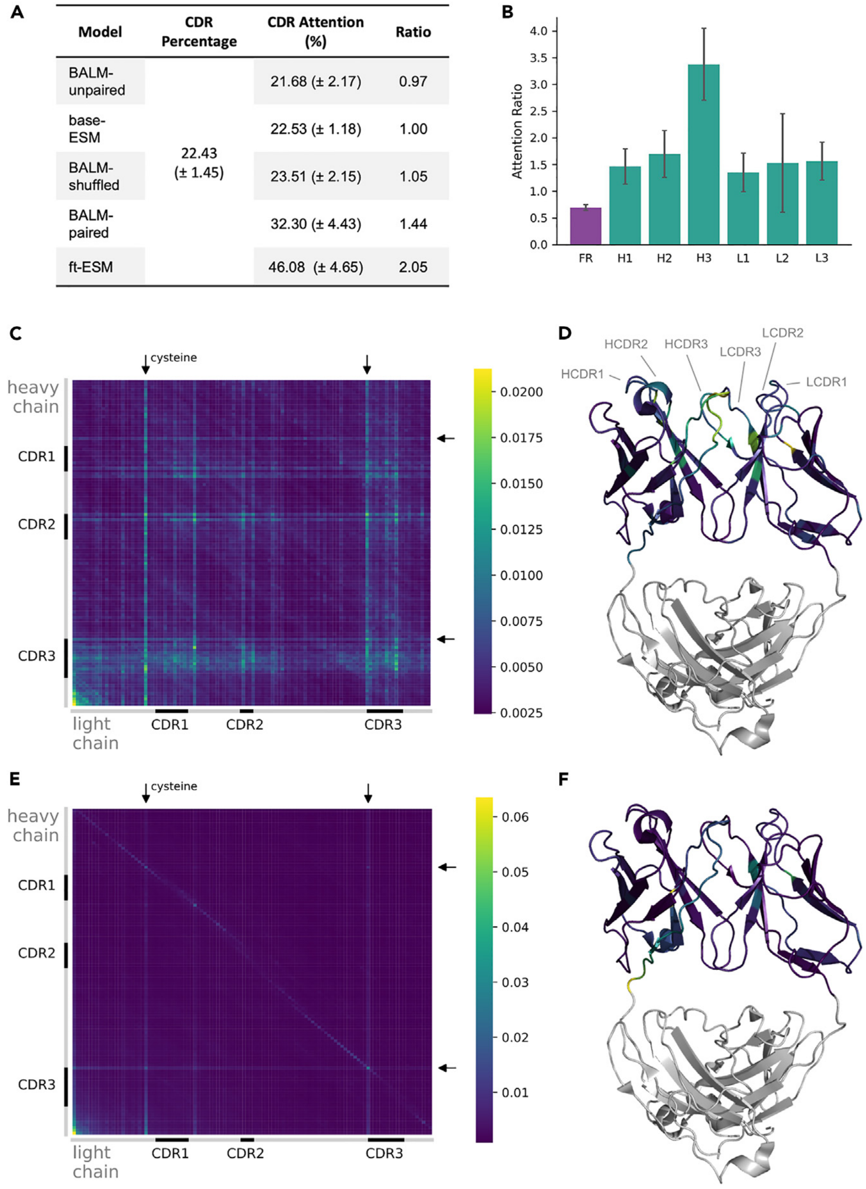

Figure 4. Cross-chain attention of ESM-2 before and after fine tuning with paired antibody sequences

(A) Cross-chain attention of the final layer of 5 models was extracted and averaged for 1,000 sequences from the test dataset, shown as mean and SD. This showed that models trained on natively paired sequences (BALM-paired and ft-ESM) show increased attention to the CDRs.

(B) Cross-chain attention for the same 1,000 sequences was plotted by CDR for ft-ESM as mean and SD, revealing that the most attention is paid to CDR-H3.

(C and E) Cross-chain attention matrices were computed for the clinically approved anti-SARS-CoV-2 mAb Masavibart by averaging cross-chain attention across all of the model layers and attention heads, using either (C) the fine-tuned ESM-2 model or (E) the base-ESM-2 model.

(D) Mapping per-position cross-chain attention of the ft-ESM-2 model onto the Masavibart structure (PDB: 6xdg) revealed a focus on structural regions important for antigen recognition.

(F) In contrast, per-position cross-chain attention of the base-ESM-2 model was focused primarily on cysteine residues and on positions near the end of the heavy chain or the start of the light chain, which are proximal in the concatenated input sequence and distant from the antigen recognition site. To demonstrate that the results observed for Masavibart are representative, cross-chain attention matrices for 4 additional anti-SARS-CoV-2 mAbs can be found in Figure S2.

during training, drives improvements in model performance. The increased cross-chain attention on CDRs is immunologically relevant because the CDRs of antibody heavy and light chains are structurally proximal and responsible for antibody function.

To further evaluate the cross-chain attention in ft-ESM, the model with the highest ratio of CDR:FR cross-chain attention, we computed the total cross-chain attention devoted to each CDR (normalized by region length) and for the combined FRs. Although more attention is paid to each of the CDRs compared to FRs, the heavy-chain CDR3 stands out as the most highly attended region (Figure 4B). This, again, is immunologically relevant because the heavy-chain CDR3 is the most diverse antibody region and is typically oriented at the interface between heavy- and light-chain variable regions. We additionally evaluated the cross-attention of several clinically approved therapeutic monoclonal antibodies (mAbs) using both the base-ESM-2 model (base-ESM) and ft-ESM. For each mAb, we extracted and averaged the cross-chain attention across all of the attention heads in all of the model layers. Results from the representative mAb Masavibart are shown in Figures 4C–4F, and data for several additional mAbs can be found in Figure S2. We observe again the extent to which ft-ESM focuses its cross-chain attention on CDRs (Figure 4C), with particular emphasis on the heavy-chain CDR3. Overlaying model attention onto the Masavibart structure reveals increased attention on regions where the heavy and light chains are in close proximity (Figure 4D). In contrast, base-ESM directs heightened attention on residues near the end of the heavy chain and the start of the light chain (lower left corner of Figure 4E), suggesting that cross-chain attention is focused on residues that are proximal in the linear input sequence rather than structurally or immunologically relevant residues (Figure 4F). Unsurprisingly for a general protein LM, base-ESM-2 also pays substantial attention to cysteine residues. This behavior appears to have transferred, albeit in a somewhat attenuated form, because ft-ESM also pays increased attention to cysteines. Because base-ESM does not preferentially attend to immunologically relevant positions before fine-tuning, these patterns in ft-ESM must be a direct result of fine-tuning with natively paired sequences.

	Classification	Model	Accuracy	F1	AUC	AUPR	MCC
A	CoV vs. Healthy Donor	BALM-unpaired	75.29 (± 0.50)	77.24 (± 0.21)	84.10 (± 0.58)	83.92 (± 0.46)	51.67 (± 0.53)
		AntiBERTy	76.77 (± 1.20)	78.18 (± 0.84)	85.30 (± 0.83)	85.08 (± 0.85)	54.03 (± 2.21)
		base-ESM	77.50 (± 1.03)	77.42 (± 0.91)	85.88 (± 0.59)	85.99 (± 0.35)	55.05 (± 2.08)
		BALM-shuffled	79.67 (± 0.42)	80.12 (± 0.60)	87.78 (± 0.41)	87.81 (± 0.36)	59.47 (± 0.89)
		BALM-paired	79.91 (± 0.51)	79.93 (± 0.53)	88.13 (± 0.27)	88.18 (± 0.27)	59.83 (± 1.02)
		ft-ESM	**81.79** (± 0.36)	**81.94** (± 0.44)	**89.72** (± 0.32)	**90.02** (± 0.22)	**63.61** (± 0.73)
B	Flu vs. CoV	BALM-unpaired	76.38 (± 1.74)	77.46 (± 1.34)	85.90 (± 1.11)	85.31 (± 1.25)	53.19 (± 3.30)
		AntiBERTy	77.47 (± 0.58)	78.97 (± 0.94)	87.58 (± 0.61)	86.56 (± 1.00)	55.92 (± 1.42)
		base-ESM	78.77 (± 1.27)	80.78 (± 0.89)	87.65 (± 0.76)	86.44 (± 0.84)	58.85 (± 2.23)
		BALM-shuffled	80.00 (± 0.69)	79.77 (± 0.68)	88.04 (± 0.95)	87.26 (± 1.05)	60.13 (± 1.37)
		BALM-paired	**82.12** (± 1.41)	**82.38** (± 1.41)	89.64 (± 1.12)	88.25 (± 1.22)	**64.28** (± 2.81)
		ft-ESM	81.30 (± 1.13)	81.01 (± 1.18)	**90.06** (± 1.19)	**88.80** (± 1.54)	62.65 (± 2.26)
C	Flu vs. CoV vs. Healthy Donor	BALM-unpaired	57.86 (± 2.18)	57.86 (± 2.17)	-	-	37.37 (± 3.04)
		AntiBERTy	61.14 (± 1.90)	60.90 (± 1.93)	-	-	41.94 (± 2.83)
		base-ESM	61.77 (± 1.13)	61.92 (± 1.05)	-	-	43.26 (± 1.49)
		BALM-shuffled	61.23 (± 0.89)	61.26 (± 0.89)	-	-	42.00 (± 1.36)
		BALM-paired	64.55 (± 1.86)	64.71 (± 1.86)	-	-	46.99 (± 2.81)
		ft-ESM	**65.59** (± 1.19)	**65.75** (± 1.20)	-	-	**48.60** (± 1.83)

D

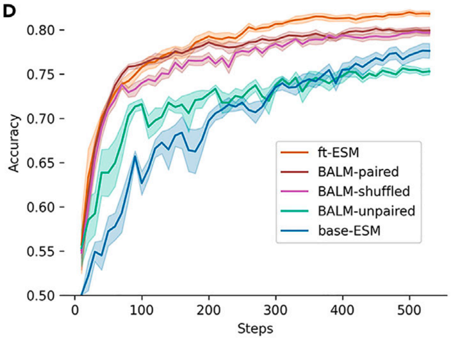

E

Model	150 steps	Final
base-ESM	68.60 (± 0.90)	77.50 (± 1.03)
BALM-unpaired	71.12 (± 0.64)	75.29 (± 0.50)
BALM-shuffled	76.71 (± 0.66)	79.67 (± 0.42)
BALM-paired	**77.75** (± 0.45)	79.91 (± 0.51)
ft-ESM	77.50 (± 0.73)	**81.79** (± 0.36)

Figure 5. Comparison of model performance on specificity classification tasks

(A) Metrics of binary classification of CoV vs. healthy donor antibodies.

(B) Metrics of binary classification of Flu vs. CoV antibodies.

(C) Metrics of multiclass classification of Flu vs. CoV vs. healthy donor antibodies.

(D) Accuracy on test dataset plotted against training steps for CoV vs. healthy donor classification.

(E) Comparison of accuracy at 150 steps vs. end of training for CoV vs. healthy donor classification. One outlier for BALM-unpaired was excluded (see Figure S3). All of the data are represented as mean and SE.

Training with natively paired antibody sequences improves specificity classification

To demonstrate an application of these natively paired models, we fine-tuned models with a sequence classification head to perform 3 separate antibody specificity classification tasks. The first task, trained on ~20,000 paired antibodies (~10,000 in each class), was a binary classification of CoV-specific antibodies against a collection of randomly selected antibodies from the memory B cell repertoires of several healthy donors. ft-ESM was the best performer across all of the metrics, followed closely by BALM-paired and then BALM-shuffled. The protein and unpaired counterparts (base-ESM and BALM-unpaired) underperform compared to their paired counterparts. AntiBERTy, a previously reported unpaired AbLM,[17] was included for comparison and appears to perform slightly better than BALM-unpaired (Figure 5A). The second task, trained on a smaller dataset of ~2,000 paired antibodies (~1,000 in each class), involved binary classification of influenza (Flu)-specific and CoV-specific antibodies. In this task, we observe similar results to the first binary classification task, with the paired models outperforming the unpaired ones, although BALM-paired outperforms ft-ESM on several metrics, unlike the first task (Figure 5B). The improved performance of all of the paired models, including BALM-shuffled, which does not learn the same immunologically relevant cross-chain features as BALM-paired or ft-ESM, suggests that at least part of the improved classification performance can be attributed to the model already being familiar with the format of paired input sequences from pretraining. This is further supported by the fact that paired models learn the classification task much faster than the unpaired ones (Figures 5D and 5E), suggesting that the initial fine-tuning with paired sequences improved the ability of the model to adapt to the specificity classification task. We see the inverse effect when fine-tuning with unpaired sequences, with unpaired models showing improved performance classifying unpaired sequences (Table S1). A notable exception is ft-ESM, which outperforms all of the other models on unpaired classification tasks, presumably due to residual familiarity with single-chain inputs remaining from its pretraining on general protein sequences.

The final specificity classification task, trained on ~3,000 antibodies (~1,000 per class), was a multiclass classification of Flu-specific, CoV-specific, and randomly selected healthy donor antibodies (Figure 5C). This is the most challenging classification and we again observe that ft-ESM and BALM-paired outperform their equivalents that lack pretraining with natively paired antibodies (BALM-unpaired and base-ESM). The performance of BALM-shuffled declines relative to the binary classification tasks, suggesting that the benefits of pretraining with natively paired sequences becomes more pronounced on increasingly difficult downstream tasks.

DISCUSSION

Many existing antibody language models are limited by their exclusive use of unpaired sequences and by inherent biases in

publicly available antibody sequence datasets, which overrepresent certain disease states. The Jaffe dataset, with $\sim 1.6 \times 10^6$ natively paired human antibody sequences from healthy donors, offers a unique opportunity to train an AbLM without these limitations. Given the relatively small size of this paired dataset, the benefits of training with natively paired sequences were not expected to overcome the shortage of data. Therefore, rather than attempting to train a state-of-the-art model using only natively paired data, we sought to determine how natively paired sequences could improve the training of AbLMs by training a matched set of models: BALM-paired, BALM-shuffled, and BALM-unpaired. In this controlled experiment, we show that natively paired training data substantially improves model performance and that these improvements are the result of BALM-paired learning immunologically relevant features that span both antibody chains in natively paired sequences.

Templated regions encoded by antibody germline segments were learned rapidly by BALM-paired, but the model struggled with nontemplated regions, including heavy-chain CDR3s and regions with increased SHM. These results suggest that model training could be improved by incorporating more somatically mutated sequences and focusing training resources on nontemplated regions. BALM-paired, BALM-shuffled, and BALM-unpaired generate informative heavy-chain embeddings that indicate their ability to learn antibody-specific features, grouping antibody embeddings primarily by mutation and secondarily by V gene use. In contrast, BALM-paired performs significantly better than BALM-shuffled and BALM-unpaired on light-chain embeddings. Although the clustered light-chain embeddings from BALM-shuffled and BALM-unpaired do not segregate into well-formed clusters, those of BALM-paired are more similar to heavy-chain embeddings, clustering primarily by mutation and secondarily by V gene. This suggests that BALM-paired is learning cross-chain features that improve light-chain embeddings that cannot be simulated with random light-chain pairing. The asymmetry with which cross-chain features influence model outputs, with light-chain embeddings displaying much more obvious differences than heavy chains, is consistent with a growing body of evidence that the light-chain partners of genetically similar heavy chains are themselves genetically similar.[21] This cross-chain information flow does not appear to be bilateral, however, because genetically similar light chains display "promiscuous" pairing with diverse heavy chains.[27] Thus, there is an immunological basis for the distinct improvement patterns observed with BALM-paired. We provide further evidence that BALM-paired is learning biologically relevant, cross-chain features by demonstrating markedly improved SHM prediction in one antibody chain when the natively paired chain, but not a germline-reverted variant of the natively paired chain, is provided as context. This implies a surprisingly sophisticated understanding of humoral immunity, learning that SHM-driven deviation from the germline template in one chain is a strong indicator of similar deviation in the paired chain.

Although these results clearly demonstrate the benefits of training antibody language models with natively paired sequence data, in practice this is less straightforward, because the cost of generating paired antibody sequences is orders of magnitude higher than that of unpaired. Our observations with BALM-shuffled suggest that datasets of synthetically paired antibody sequences are unlikely to be useful for AbLM training unless they accurately recapitulate all of the factors that influence native antibody pairing. To evaluate the feasibility of a middle-ground approach in which paired antibody sequences are used to supplement larger and more readily available training datasets, we fine-tuned the general protein language model ESM-2[14] using the Jaffe dataset (ft-ESM). Using this model, along with our previous BALM models, we further assessed the cross-chain features being learned by the model. We observed that the natively paired models (ft-ESM and BALM-paired) showed increased attention to the CDRs, compared to the unpaired models (BALM-unpaired and base-ESM) and the randomly paired model (BALM-shuffled). The focused attention on the CDRs shows focus on the immunologically important and structurally proximal regions of paired antibody sequences. The fact that ft-ESM shows more CDR attention than BALM-paired is particularly encouraging because it indicates that natively paired datasets, which due to their high cost are necessarily limited in scale, can be supplemented with unpaired antibody sequences or general protein sequences while still allowing models to learn critically important cross-chain antibody features.

To demonstrate an example application of these paired models, we trained sequence classifiers to test the ability of the models to perform 3 separate antibody specificity classification tasks. We observed that the natively paired models, ft-ESM and BALM-paired, consistently outperformed their counterparts base-ESM and BALM-unpaired. BALM-shuffled also outperforms the unpaired models on the binary classification tasks, performing only slightly below the natively paired models. However, on the 3-way classification task, BALM-shuffled performs lower than the natively paired models (more comparably with the unpaired models), suggesting that the cross-chain features learned from natively paired sequences is more significant for complex downstream tasks. We also observed that ft-ESM outperforms other models across paired classification tasks and even on unpaired classification tasks (where unpaired models tend to dominate), suggesting that ft-ESM is more flexible with the types of data during downstream tasks. This implies that mixed models trained on a mix of paired and unpaired or protein sequences may be a useful strategy both to overcome the shortage of natively paired data and to ensure the generalizability of pretrained models.

Although the results from these binary and small multiclass classification tasks are impressive, it is not clear whether there is much practical use for models that can perform relatively simple "SARS-CoV-2 or not" classification tasks. Instead, the fact that these models can achieve such high accuracy means that there are learnable patterns of sequence-inherent properties that distinguish groups of antibodies with similar specificity. Herein lies what is likely the greatest value of these models: if we can better understand the feature patterns driving classification decisions and leverage these patterns in other downstream tasks, then we have the opportunity to learn the fundamental immunological properties that define antibody specificity, with broad ramifications across infectious disease, autoimmunity, and cancer.

In summary, we report four important discoveries that will help guide the design and training of future state-of-the-art AbLMs. First, incorporating natively paired training data produces

higher-performing models by allowing models to learn cross-chain features that cannot be simulated by randomly paired sequences. The native pairing of heavy and light chains is integral to the structure and function of each antibody and understanding features that span both chains is vital. Second, AbLMs rapidly learn patterns associated with templated regions that are encoded by germline gene segments but struggle with nontemplated regions. It is likely that training datasets enriched in somatically mutated sequences combined with antibody-specific training schemes that bias training resources toward untemplated regions such as CDR3s may directly address the most prominent model weaknesses. Third, mixed training datasets, which supplement paired antibody sequences with unpaired or general protein data, can help overcome the high cost and limited availability of natively paired datasets. Finally, LMs trained or fine-tuned using natively paired antibody sequences perform better on downstream classification tasks, suggesting a deeper and more generalizable understanding of human antibodies.

EXPERIMENTAL PROCEDURES

Resource availability

Lead contact

Further information and requests for resources should be directed to and will be fulfilled by the lead contact, Bryan Briney (briney@scripps.edu).

Materials availability

This study did not generate new unique reagents.

Data and code availability

Model weights for BALM-paired, BALM-shuffled, BALM-unpaired, and ft-ESM and the datasets used for training are available on Zenodo[28] under the CC BY-SA 4.0 license. The code used for data processing, model training, and cross-chain attention plots is available on Github (github.com/brineylab/BALM-paper) under the MIT license and has also been archived to Zenodo together with the model weights and training datasets. Any other information required to reanalyze the data reported is available from the lead contact upon request.

Training data

For BALM-paired pretraining, we used the largest publicly available dataset of natively paired human antibody sequences, comprising $\sim 1.6 \times 10^6$ sequence pairs.[21,22] All paired antibody sequences in this dataset were recovered from circulating B cells from healthy adult human donors and were not selected or enriched for binding to any particular antigen. Raw sequences were annotated with abstar,[29] and the amino acid sequence of each V(D)J region was extracted. Sequence pairs were filtered to remove duplicates and nonproductive sequences, resulting in 1,335,854 filtered pairs. A total of 90% of the filtered pairs were used for training, with 5% held out for evaluation and an additional 5% for testing.

To generate the shuffled-pairs dataset for BALM-shuffled, the heavy and light chains from the BALM-paired dataset were randomly shuffled. Due to the redundancy of light chains, a very small percentage (0.07%, or 845 sequences) of the pairs in the train dataset after shuffling were native pairs.

To generate the unpaired dataset for BALM-unpaired, the BALM-paired dataset was processed to unpair the sequences. This separation of pairs occurred using the train-evaluation-testing split from BALM-paired, such that the training corpus of BALM-paired and BALM-unpaired are directly comparable. The unpaired data were intentionally not processed any further to ensure that the models were trained on the same sequence data and therefore were directly comparable. This means, however, that given that light chains have less diversity than heavy chains, there is a high level of light-chain redundancy in the unpaired dataset. Out of the 2,671,708 total sequences, 746,311 of the light chains were redundant, meaning there are a total of 1,925,397 unique sequences in the unpaired dataset. This light-chain redundancy disadvantages BALM-unpaired during training; however, it was an intentional choice to include this light-chain redundancy because the paired models also see these redundant light chains but with the advantage of its heavy-chain pair.

For specificity classification training, 3 datasets were used. CoV antibody sequences were obtained from CoV-AbDab.[30] Flu antibody sequences were obtained from Wang et al.,[31] filtered for paired sequences only. Randomly selected antibodies from the memory B cell repertoire of healthy adult donors were obtained from the control dataset of Hurtado et al.[32] Amino acid sequences were clustered at 95% identity for CoV vs. healthy donor and 99% for the other two classification tasks. From here, these datasets were used to form 3 unique datasets to use for specificity classification tasks and labeled according to their antigen specificity: CoV vs. healthy donor (total 18,090 sequences), CoV vs. Flu (total 2,930 sequences), and CoV vs. Flu vs. healthy donor (total 4,396 sequences). Sequences were labeled according to their antigen specificity (or nonspecificity, for the healthy donor sequences), and each dataset contained an equal number of each class to ensure balanced training. All 3 datasets were randomly split with stratification, to generate a test dataset of 5% for CoV vs. healthy donor and 10% for the other 2 tasks.

BALM training

We separately trained 3 BALM variants, BALM-paired, BALM-shuffled, and BALM-unpaired, using the HuggingFace transformers library.[33] All 3 models used a slightly modified version of the RoBERTa-large architecture,[34] with 24 layers, 16 attention heads per layer, a hidden size of 1,024, and an intermediate (feedforward) size of 4,096. An encoder-only architecture was chosen to align with other widely used protein and antibody models and prioritize utility for downstream tasks such as specificity classification. In addition, absolute positional embeddings were selected over rotary embeddings to increase the compute efficiency of model training. An MLM objective was selected rather than another pretraining method, such as Electra's replaced token detection,[35] since ProtBERT has been previously shown to outperform ProtElectra on general protein tasks.[36]

The vocabulary contained 25 tokens: 1 for each of the 20 amino acids and 5 special tokens: <s>, </s>, <pad>, <unk>, and <mask>. Inputs to BALM-unpaired were individual heavy- or light-chain sequences, padded to a maximum length of 256, to accommodate the longest unpaired sequence in the dataset without truncation. Inputs to BALM-paired were concatenated heavy- and light-chain sequences separated by a </s> token and padded to a maximum input length of 512, such that the input length was twice that of BALM-unpaired. Since BALM-unpaired has twice as many sequences as BALM-paired, the total batch size of BALM-unpaired (512) was twice that of BALM-paired (256) to normalize training.

All 3 models were trained using an MLM objective. Briefly, when given an input for which some positions have been masked, the model is asked to predict the masked tokens based only on the context provided by the non-masked tokens. For each input, 15% of the tokens were uniformly selected for masking. Of the selected tokens, 80% were replaced with a <mask> token, 10% were replaced with a randomly selected amino acid token, and 10% were left unchanged. Masking was performed dynamically to avoid using the same mask across epochs.[34] The 3 models were each trained for 500,000 steps ($\sim$100 epochs) on 8 NVIDIA A100 graphics processing units (GPUs), which equates to $\sim$5 days per model. The peak learning rate was $4e{-}4$, with a linear warmup over the first 30,000 steps and a linear decay thereafter.

Analysis of model embeddings

The output embedding of a model with input length L, hidden size H, and N input sequences, is a matrix of the shape $N \times H \times L$. For each BALM model, the dimensionality of the final layer output embedding was reduced by averaging over the L dimension as previously described,[16,18] producing an $N \times H$ matrix. A UMAP embedding[25] was computed for the averaged embeddings matrix for each model in Python 3.9, using the umap-learn package.[26] UMAP plots were visualized in Python 3.9 using matplotlib. For BALM-paired and BALM-shuffled, the subset of the output embedding matrix corresponding to either the heavy chain or light chain was extracted before averaging so that only the embeddings for the chain of interest were used to compute the UMAP. This ensures an "apples-to-apples" comparison between the embeddings of BALM-paired and BALM-shuffled (for which the raw embeddings contain both heavy and light chains) and BALM-unpaired (for which the raw

embeddings contain only a single chain). The same procedure was completed for the t-SNE embeddings in Figure S1.

ft-ESM training

We fine-tuned the pretrained 650-million parameter ESM-2 model, which is based on the RoBERTa architecture[34] and has 33 layers, with 20 attention heads per layer.[14] The 650-million parameter model was chosen (rather than the larger, higher-performing 3 or 15 billion parameter ESM-2 variants) to reduce the likelihood of overfitting due to the small training dataset and allow for faster training despite memory constraints. Inputs were concatenated heavy- and light-chain sequences separated by two <cls> tokens and were tokenized with the standard ESM-2 vocabulary and padded to a maximum length of 320. None of the paired or unpaired sequences exceeded the maximum input length, so truncation was not required. The total batch size was 256. The model was trained using an MLM objective, as described above for BALM model training. The peak learning rate was 4e−4, with a linear warmup over the first 30,000 steps and a linear decay thereafter. The model was scheduled to train for 500,000 steps on 8 NVIDIA A100 GPUs, but was early-stopped after 150,000 steps to prevent overfitting being observed in the evaluation dataset, which equates to ~7 days.

Analysis of cross-chain attention

Attention values of the final layer of each model were extracted for each position of the input antibody sequence and filtered to include only cross-attention (i.e., position pairs for which the 2 positions are on different chains). Values were averaged by position in the sequences, and then analyzed by their location (FR or CDR) in the sequence. To generate the attention ratio, the percentage of attention to the CDRs was divided by the percentage of the sequence classified as CDR positions. These values were also plotted into bar plots by CDR group using seaborn[37] and matplotlib.[38]

For the therapeutic antibodies, the attention values of ft-ESM and base-ESM were extracted for each position of the input antibody sequence, across each head and layer of the model. Cross-attention values for each position pair were averaged across all 20 heads and 33 layers of the model. Based on these data, heatmaps were generated using seaborn[37] and matplotlib.[38] To map the cross-chain attention onto mAb structures, the total cross-chain attention was separately summed for each position in the heavy and light chains, resulting in a single attention vector per chain. These attention vectors were used to color residues by b-factor using PyMOL.[39] Attention step plots were created using the summed attention vectors in Python using matplotlib.[38]

Specificity classification training

Models were fine-tuned with a sequence classification head for the downstream task of specificity prediction on two binary classifications (CoV vs. healthy donor, CoV vs. Flu) and one multiclass classification (CoV vs. Flu vs. healthy donor). For tokenization, models were tokenized with the standard tokenizer for the model type. BALM models received concatenated heavy- and light-chain sequences separated by the </s> token, whereas ESM models were concatenated heavy- and light-chain sequences separated by 2 <cls> tokens. No truncation was necessary since all of the sequences were shorter than the maximum input length of the model. Models were trained for 1 epoch with a total batch size of 32 for CoV vs. healthy donor and 8 for the other 2 classifications, with a learning rate of 5e−5 and a linear warmup ratio of 0.1. Each model was trained for each sequence classification task 5 times, with the same 5 random dataset splits and different random seeds during training, to show variation based on training data and random seeds.

Metrics used for evaluation of the binary classifications were accuracy, F1, area under the receiver operating characteristic curve (AUC), area under the precision-recall curve (AUPR), and Matthews correlation coefficient (MCC). For the multiclass classifications, evaluation metrics were accuracy, macro-F1, and MCC. Plot of accuracy against model steps for healthy donor vs. CoV was based on wandb logging data, averaged across all 5 runs of each model with SE, and plots were smoothed with a weight of 0.25. One iteration of BALM-unpaired was excluded as an outlier and rerun with a different random seed, and the training plot for the excluded outlier can be viewed in Figure S3.

SUPPLEMENTAL INFORMATION

Supplemental information can be found online at https://doi.org/10.1016/j.patter.2024.100967.

ACKNOWLEDGMENTS

The authors thank Wyatt McDonnell for thoughtful discussions and helpful feedback on early manuscript drafts. This work was funded by the National Institutes of Health awards R35-GM133682, P01-AI177683, U19-AI135995, R01-AI171438, P30-AI036214, and UM1-AI144462.

AUTHOR CONTRIBUTIONS

Conceptualization: S.B. and B.B. Code: S.B. and B.B. Model training and evaluation: S.B. and B.B. Manuscript preparation: S.B. and B.B. Manuscript revision: S.B. and B.B.

DECLARATION OF INTERESTS

B.B. is an equity shareholder in Infinimmune and a member of their Scientific Advisory Board.

Received: November 9, 2023
Revised: January 25, 2024
Accepted: March 8, 2024
Published: March 29, 2024

REFERENCES

1. Briney, B., Inderbitzin, A., Joyce, C., and Burton, D.R. (2019). Commonality despite exceptional diversity in the baseline human antibody repertoire. Nature *566*, 393–397. https://doi.org/10.1038/s41586-019-0879-y.
2. Mora, C., Tittensor, D.P., Adl, S., Simpson, A.G.B., and Worm, B. (2011). How many species are there on Earth and in the ocean? PLoS Biol. *9*, e1001127. https://doi.org/10.1371/journal.pbio.1001127.
3. Tonegawa, S. (1983). Somatic generation of antibody diversity. Nature *302*, 575–581. https://doi.org/10.1038/302575a0.
4. MacLennan, I.C. (1994). Germinal centers. Annu. Rev. Immunol. *12*, 117–139. https://doi.org/10.1146/annurev.iy.12.040194.001001.
5. Muramatsu, M., Sankaranand, V.S., Anant, S., Sugai, M., Kinoshita, K., Davidson, N.O., and Honjo, T. (1999). Specific expression of activation-induced cytidine deaminase (AID), a novel member of the RNA-editing deaminase family in germinal center B cells. J. Biol. Chem. *274*, 18470–18476. https://doi.org/10.1074/jbc.274.26.18470.
6. Victora, G.D., and Nussenzweig, M.C. (2012). Germinal centers. Annu. Rev. Immunol. *30*, 429–457. https://doi.org/10.1146/annurev-immunol-020711-075032.
7. Mesin, L., Ersching, J., and Victora, G.D. (2016). Germinal Center B Cell Dynamics. Immunity *45*, 471–482. https://doi.org/10.1016/j.immuni.2016.09.001.
8. McHeyzer-Williams, M., Okitsu, S., Wang, N., and McHeyzer-Williams, L. (2011). Molecular programming of B cell memory. Nat. Rev. Immunol. *12*, 24–34. https://doi.org/10.1038/nri3128.
9. Seifert, M., and Küppers, R. (2016). Human memory B cells. Leukemia *30*, 2283–2292. https://doi.org/10.1038/leu.2016.226.
10. Anfinsen, C.B. (1973). Principles that Govern the Folding of Protein Chains. Science *181*, 223–230. https://doi.org/10.1126/science.181.4096.223.
11. Bepler, T., and Berger, B. (2021). Learning the protein language: Evolution, structure, and function. Cell Syst. *12*, 654–669.e3. https://doi.org/10.1016/j.cels.2021.05.017.
12. Rives, A., Meier, J., Sercu, T., Goyal, S., Lin, Z., Liu, J., Guo, D., Ott, M., Zitnick, C.L., Ma, J., and Fergus, R. (2021). Biological structure and function emerge from scaling unsupervised learning to 250 million protein

CellPress
OPEN ACCESS

sequences. Proc. Natl. Acad. Sci. USA *118*, e2016239118. https://doi.org/10.1073/pnas.2016239118.

13. Fang, X., Wang, F., Liu, L., He, J., Lin, D., Xiang, Y., Zhang, X., Wu, H., Li, H., and Song, L. (2022). HelixFold-Single: MSA-free Protein Structure Prediction by Using Protein Language Model as an Alternative. Preprint at arXiv. https://doi.org/10.48550/arXiv.2207.13921.
14. Lin, Z., Akin, H., Rao, R., Hie, B., Zhu, Z., Lu, W., Smetanin, N., Verkuil, R., Kabeli, O., Shmueli, Y., et al. (2023). Evolutionary-scale prediction of atomic-level protein structure with a language model. Science *379*, 1123–1130. https://doi.org/10.1126/science.ade2574.
15. Choi, Y. (2022). Artificial intelligence for antibody reading comprehension: AntiBERTa. Patterns *3*, 100535. https://doi.org/10.1016/j.patter.2022.100535.
16. Olsen, T.H., Moal, I.H., and Deane, C.M. (2022). AbLang: an antibody language model for completing antibody sequences. Bioinform. Adv. *2*, vbac046. https://doi.org/10.1093/bioadv/vbac046.
17. Ruffolo, J.A., Gray, J.J., and Sulam, J. (2021). Deciphering antibody affinity maturation with language models and weakly supervised learning. Preprint at arXiv. https://doi.org/10.48550/arXiv.2112.07782.
18. Leem, J., Mitchell, L.S., Farmery, J.H.R., Barton, J., and Galson, J.D. (2022). Deciphering the language of antibodies using self-supervised learning. Patterns *3*, 100513. https://doi.org/10.1016/j.patter.2022.100513.
19. Kovaltsuk, A., Leem, J., Kelm, S., Snowden, J., Deane, C.M., and Krawczyk, K. (2018). Observed Antibody Space: A Resource for Data Mining Next-Generation Sequencing of Antibody Repertoires. J. Immunol. *201*, 2502–2509. https://doi.org/10.4049/jimmunol.1800708.
20. Olsen, T.H., Boyles, F., and Deane, C.M. (2022). Observed Antibody Space: A diverse database of cleaned, annotated, and translated unpaired and paired antibody sequences. Protein Sci. *31*, 141–146. https://doi.org/10.1002/pro.4205.
21. Jaffe, D.B., Shahi, P., Adams, B.A., Chrisman, A.M., Finnegan, P.M., Raman, N., Royall, A.E., Tsai, F., Vollbrecht, T., Reyes, D.S., et al. (2022). Functional antibodies exhibit light chain coherence. Nature *611*, 352–357. https://doi.org/10.1038/s41586-022-05371-z.
22. Jaffe, D.B., Shahi, P., Adams, B.A., Chrisman, A.M., Finnegan, P.M., Raman, N., Royall, A.E., Tsai, F., Vollbrecht, T., Reyes, D.S., et al. (2022). Functional antibodies exhibit light chain coherence. Zenodo. https://doi.org/10.5281/zenodo.6348137.
23. Alberts, B., Johnson, A., Lewis, J., Raff, M., Roberts, K., and Walter, P. (2002). The Generation of Antibody Diversity (Garland Science).
24. Hofmann, H., Wickham, H., and Kafadar, K. (2017). Letter-Value Plots: Boxplots for Large Data. J. Comput. Graph Stat. *26*, 469–477. https://doi.org/10.1080/10618600.2017.1305277.
25. Becht, E., McInnes, L., Healy, J., Dutertre, C.-A., Kwok, I.W.H., Ng, L.G., Ginhoux, F., and Newell, E.W. (2018). Dimensionality reduction for visualizing single-cell data using UMAP. Nat. Biotechnol. *37*, 38–44. https://doi.org/10.1038/nbt.4314.
26. McInnes, L., Healy, J., Saul, N., and Großberger, L. (2018). UMAP: Uniform Manifold Approximation and Projection. J. Open Source Softw. *3*, 861. https://doi.org/10.21105/joss.00861.
27. DeKosky, B.J., Kojima, T., Rodin, A., Charab, W., Ippolito, G.C., Ellington, A.D., and Georgiou, G. (2015). In-depth determination and analysis of the human paired heavy- and light-chain antibody repertoire. Nat. Med. *21*, 86–91. https://doi.org/10.1038/nm.3743.
28. Burbach, S., and Briney, B. (2023). Improving antibody language models with native pairing. Zenodo. https://doi.org/10.5281/zenodo.10684811.
29. Briney, B., and Burton, D.R. (2018). Massively scalable genetic analysis of antibody repertoires. Preprint at bioRxiv. https://doi.org/10.1101/447813.
30. Raybould, M.I.J., Kovaltsuk, A., Marks, C., and Deane, C.M. (2021). CoV-AbDab: the coronavirus antibody database. Bioinformatics *37*, 734–735. https://doi.org/10.1093/bioinformatics/btaa739.
31. Wang, Y., Lv, H., Lei, R., Yeung, Y.-H., Shen, I.R., Choi, D., Teo, Q.W., Tan, T.J.C., Gopal, A.B., Chen, X., et al. (2023). An explainable language model for antibody specificity prediction using curated influenza hemagglutinin antibodies. Preprint at bioRxiv. https://doi.org/10.1101/2023.09.11.557288.
32. Hurtado, J., Rogers, T.F., Jaffe, D.B., Adams, B.A., Bangaru, S., Garcia, E., Capozzola, T., Messmer, T., Sharma, P., Song, G., et al. (2023). Deep repertoire mining uncovers ultra-broad coronavirus neutralizing antibodies targeting multiple spike epitopes. Preprint at bioRxiv. https://doi.org/10.1101/2023.03.28.534602.
33. Wolf, T., Debut, L., Sanh, V., Chaumond, J., Delangue, C., Moi, A., Cistac, P., Rault, T., Louf, R., Funtowicz, M., et al. (2019). HuggingFace's Transformers: State-of-the-art Natural Language Processing. Preprint at arXiv. https://doi.org/10.48550/arXiv.1910.03771.
34. Liu, Y., Ott, M., Goyal, N., Du, J., Joshi, M., Chen, D., Levy, O., Lewis, M., Zettlemoyer, L., and Stoyanov, V. (2019). RoBERTa: A Robustly Optimized BERT Pretraining Approach. Preprint at arXiv. https://doi.org/10.48550/arXiv.1907.11692.
35. Clark, K., Luong, M.-T., Le, Q.V., and Manning, C.D. (2020). ELECTRA: Pre-training Text Encoders as Discriminators Rather Than Generators. Preprint at arXiv. https://doi.org/10.48550/arXiv.2003.10555.
36. Elnaggar, A., Heinzinger, M., Dallago, C., Rehawi, G., Wang, Y., Jones, L., Gibbs, T., Feher, T., Angerer, C., Steinegger, M., et al. (2022). ProtTrans: Toward Understanding the Language of Life Through Self-Supervised Learning. IEEE Trans. Pattern Anal. Mach. Intell. *44*, 7112–7127. https://doi.org/10.1109/TPAMI.2021.3095381.
37. Waskom, M. (2021). seaborn: statistical data visualization. J. Open Source Softw. *6*, 3021. https://doi.org/10.21105/joss.03021.
38. Hunter, J.D. (2007). Matplotlib: A 2D Graphics Environment. Comput. Sci. Eng. *9*, 90–95. https://doi.org/10.1109/MCSE.2007.55.
39. Schrödinger, L.L.C., and DeLano, W. (2020). PyMOL. https://github.com/schrodinger/pymol-open-source.

Patterns, Volume *5*

Supplemental information

Improving antibody language models with native pairing

Sarah M. Burbach and Bryan Briney

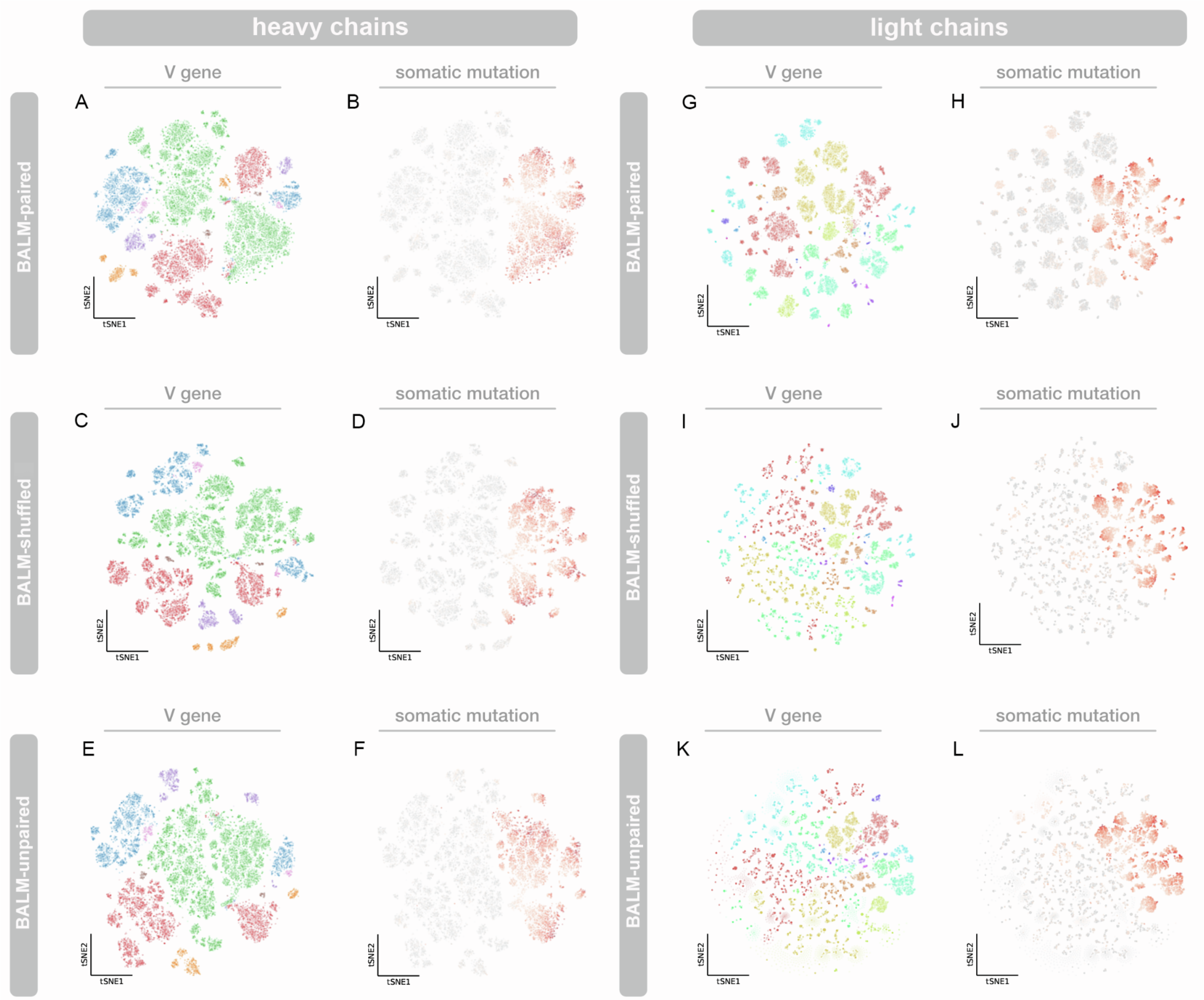

Figure S1. tSNE shows improved clustering of light chains with natively paired sequences. tSNE of final layer embeddings for heavy chains (A-F) and light chains (G-L), colored by V-gene or number of somatic mutations for BALM-paired, BALM-shuffled, and BALM-unpaired. We observe comparable results to the UMAP shown in Figure 2, with v-genes clustering of unmutated light chains being improved by the native pairing in BALM-paired, compared to the more random v-gene clustering observed in BALM-shuffled and BALM-unpaired.

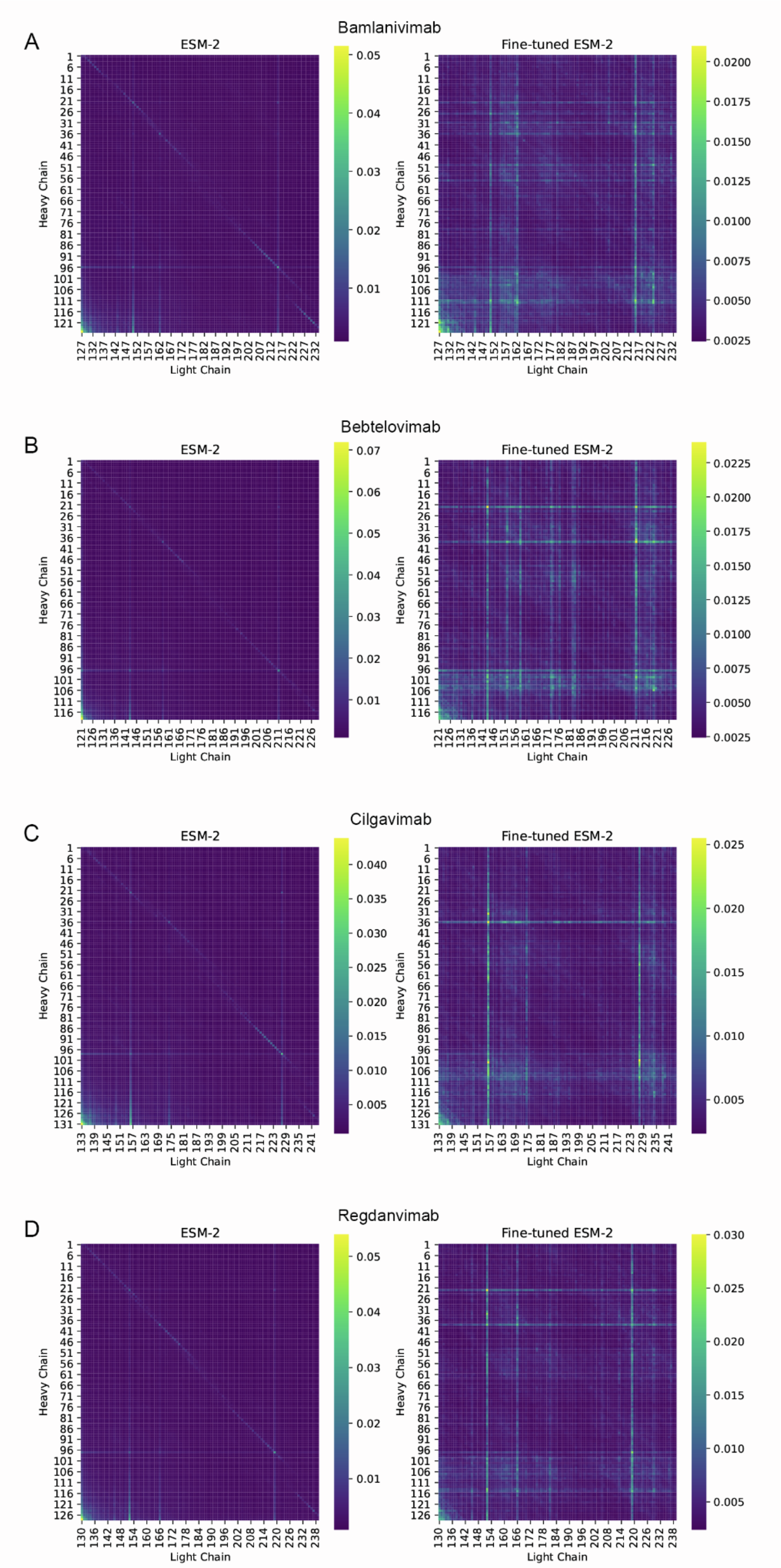

Figure S2. Cross-chain attention for selected therapeutic mAbs. Four therapeutic mAbs against SARS-CoV-2 were processed using base-ESM (right plots) or ft-ESM (left plots): Bamlanivimab (A), Bebtelovimab (B), Cilgavimab (C), and Regdanvimab (D). Cross-chain attention was computed by averaging attention from all heads of each model layer.

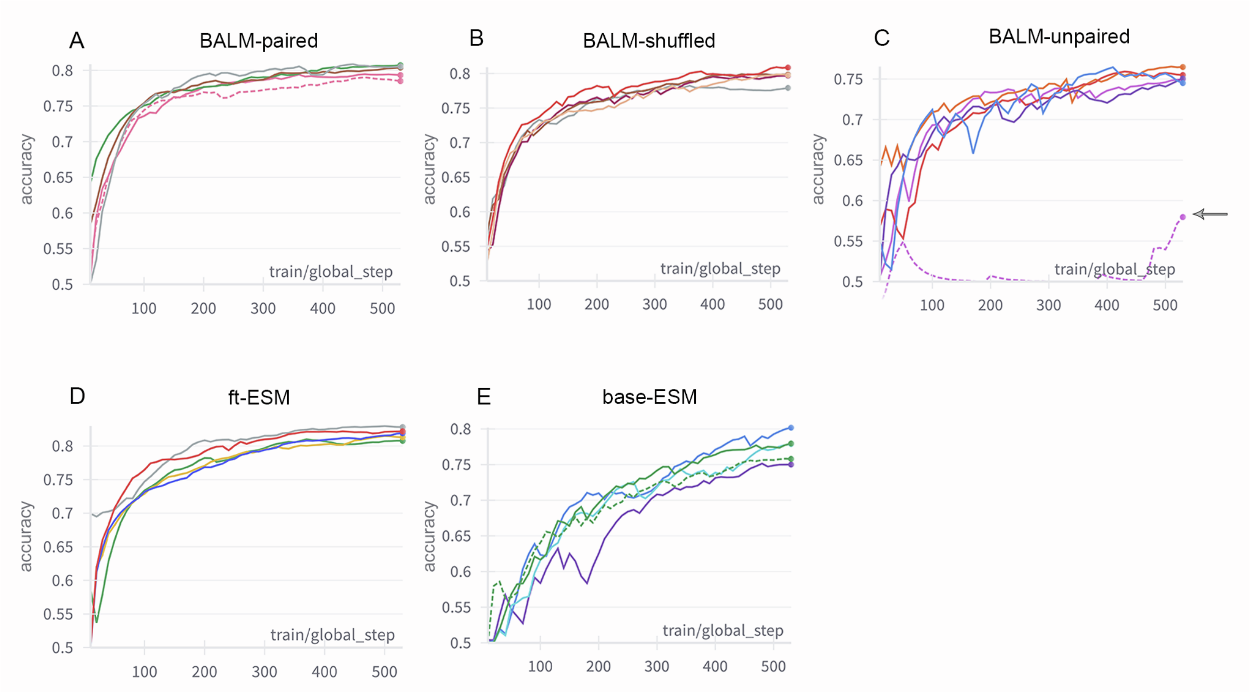

Figure S3. Accuracy training plots for HD vs CoV sequence classification task. Shows the training plots (plotting accuracy vs training steps) for HD vs CoV classification for all five models. We note with an arrow the outlier in BALM-unpaired (C) that was excluded and re-ran with the same dataset, but a different random seed, for Figure 5D.

Table S1. Unpaired classification results for HD vs CoV classification task. Shows results for classification task with the same datasets as the paired HD vs CoV task in Figure 5A, with only heavy chains provided. We observe that model performance of the unpaired / protein models increases and model performance of the paired models decreases compared to the paired classification task. Despite this, ft-ESM remains the highest-performing model, suggesting that mixed-training models are more flexible about the types of data they can accommodate, and therefore are better suited downstream tasks with unpaired datasets than models trained exclusively on paired sequences.

Classification	Model	Accuracy	F1	AUC	AUPR	MCC
CoV vs. Healthy Donor Unpaired Sequences	base-ESM	76.00 (± 0.68)	76.44 (± 0.53)	83.91 (± 0.59)	84.28 (± 0.60)	52.06 (± 1.33)
	BALM-paired	76.66 (± 0.69)	77.12 (± 0.53)	84.84 (± 0.39)	84.90 (± 0.31)	53.45 (± 1.34)
	BALM-unpaired	77.04 (± 0.60)	76.52 (± 0.65)	85.82 (± 0.48)	86.09 (± 0.48)	54.15 (± 1.20)
	BALM-shuffled	77.41 (± 0.90)	78.12 (± 0.79)	85.19 (± 0.89)	85.32 (± 0.86)	55.06 (± 1.77)
	AbLang-H	77.99 (± 0.44)	78.13 (± 0.52)	86.49 (± 0.49)	86.38 (± 0.43)	56.02 (± 0.88)
	AntiBERTy	78.08 (± 0.61)	78.80 (± 0.56)	85.80 (± 0.42)	85.53 (± 0.35)	56.32 (± 1.20)
	ft-ESM	**78.72** (± 0.65)	**78.96** (± 0.45)	**86.72** (± 0.48)	**87.09** (± 0.31)	**57.49** (± 1.26)
